# Eye-tracking does not reveal early attention processing of sexual copulatory movement in heterosexual men and women

**DOI:** 10.1038/s41598-024-53243-5

**Published:** 2024-03-04

**Authors:** Ondřej Vaníček, Lucie Krejčová, Martin Hůla, Kateřina Potyszová, Kateřina Klapilová, Klára Bártová

**Affiliations:** 1 Department of Psychology and Life Sciences, Faculty of Humanities, Charles University, Pátkova 2137/5, 182 00 Prague, Czech Republic; 2 Present Address: Center for Sexual Health and Interventions, National Institute of Mental Health, Topolová 748, 250 67 Klecany, Czech Republic

**Keywords:** Human behaviour, Sexual behaviour, Attention

## Abstract

Men and women respond differently when presented with sexual stimuli. Men's reaction is gender-specific, and women's reaction is gender-nonspecific. This might be a result of differential cognitive processing of sexual cues, namely copulatory movement (CM), which is present in almost every dynamic erotic stimulus. A novelty eye-tracking procedure was developed to assess the saliency of short film clips containing CM or non-CM sexual activities. Results from 29 gynephilic men and 31 androphilic women showed only small and insignificant effects in attention bias and no effects in attentional capture. Our results suggest that CM is not processed differently in men and women and, therefore, is not the reason behind gender-nonspecific sexual responses in women.

## Introduction

There is a striking difference in genital responses to erotic stimuli between gynephilic (sexually attracted to adult females) men and androphilic (sexually attracted to adult males) women. A considerable body of research demonstrates that men tend to react with sexual arousal exclusively to sexual cues concordant with their sexual preferences. This kind of response pattern has been termed as gender-specific^1^. In contrast, women's sexual arousal tends to be rather gender-nonspecific, meaning that they respond with sexual genital arousal to stimuli containing even non-preferred sexual content^2^.

In the first study to experimentally explore this line of research, Chivers et al.^3^ presented gynephilic and androphilic men and women as well as transgender women (assigned male at birth) with erotic videos of heterosexual, lesbian, or gay pairs. Men of both orientations and transgender women showed decisively gender-specific reactions, with gynephilic individuals having strong genital responses to heterosexual and lesbian films and androphilic individuals having strong genital responses to gay films. Women’s genital response, on the other hand, did not differentiate between the films while still being larger than response to neutral stimulus. Such response patterns were mirrored—to a lesser extent—in participants’ evaluations of subjective arousal. These results were later replicated in a follow-up study^4^ on a relatively small sample of gynephilic men and androphilic women. Curiously, women also demonstrated some levels of genital arousal in response to a video depicting copulating primates. Similar findings were later reported in a similar study with both gynephilic and androphilic samples of men and women^5^. A study published the following year by Suschinsky et al.^6^ demonstrated greater gender-specificity in genital responses of gynephilic men compared to androphilic women. In the next two studies reported by Peterson et al.^7^, both androphilic and gynephilic women showed gender-nonspecific patterns of genital response. In 2014, an unexpected finding was reported in the study by Spape et al.^8^. Androphilic women exhibited gender-specific patterns of both subjective and genital responses when presented with pictures of erect penises and exposed vulvas. This was the first study to demonstrate gender-specific genital responses in androphilic women, but it was not the only one. Four years later, other researchers reported on gender-specific reactions in androphilic women when viewing erotic film clips, but only when their responses were measured via vaginal lubrication assessment. When measured with more traditional VPG (vaginal plethysmography measuring vaginal vasocongestion), the results were consistent with previous nonspecific findings^9^. This gender-nonspecificity is reflected in women’s cognitive processing of sexual stimuli (e.g.^10–12^). Studies using an eye-tracking device revealed that the gender-specificity of gazing patterns was less pronounced in women than in men when viewing sexual stimuli^13–15^. Overall, throughout the majority of studies, female sexual responses follow a gender-nonspecific pattern.

Several hypotheses try to explain this phenomenon. For example, female sexuality might be more malleable by external influences such as social, cultural, and other contextual factors^16^. The recent cross-cultural eye-tracking study suggests that women’s ratings and gazing patterns are more shaped by culture than men’s ratings^17^. Another hypothesis suggests that in a sexual context, an automatic genital response occurs as a protective mechanism to prevent pain and injury during potential vaginal penetration^18^. Such a hypothesis is, however, contradicted by gender-specific responses found when assessing vaginal lubrication as a measure of female genital arousal, as described earlier^9^. Women may also identify with the sexual pleasure of an individual depicted by the erotic stimulus^1^. While giving different answers to similar findings, all of these hypotheses deal with how the sexual stimuli are cognitively processed, specifically focusing on culturally conditioned sexual cues, universal sexual cues, or cues specifically related to witnessed sexual pleasure. The Information Processing Model by Janssen et al.^19^ provides a theoretical framework for understanding the specificity of sexual response among women and men. It highlights the importance of attention to sexual cues as a key component of sexual response. Sexual arousal emerges in response to the activation of sexual meaning in reference to memory connected with the automatic genital response. The attentional system then enhances the activation of sexual meaning through subjective experiences associated with the stimulus. The stimulus features play a key role in this process, with selected sexual cues capturing attention faster and sustaining it longer during the stimulus processing^20,21^. It is the selection of particular sexual cues that leads to a classification of stimulus as sexually relevant and ultimately to the emergence of a genital response^22^. Previous research has found that predictions regarding the gender-specificity of women’s early visual attention are unsupported for androphilic women. In contrast, gender-specific effects are shown for both gynephilic and androphilic men and gynephilic women. Moreover, it was found that androphilic women show gender-nonspecificity in early stimulus processing and more gender-specificity in later stimulus processing, while gynephilic women show gender-specificity at most stages of sexual response (for review, see^1^).

The theoretical model by Janssen et al.^19^ gives no exact answer as to what constitutes a sexually relevant cue. However, some clues might be deduced from the existing body of research. For example, while watching sexually loaded videos, men’s sexual arousal was mainly dependent upon the perceived attractiveness of the female actress^23^. There was no distinct feature of the films that elicited stronger sexual arousal in women but their ability to identify with the actress. However, these gender differences were not compared directly since the analysis was done separately for men and women. Dawson and Chivers^24^ employed an eye-tracking methodology to explore how stimulus modality (static or dynamic) and stimulus features, such as gender, sexual activity, and nonsexual contextual cues, influence attentional processing of sexual stimuli in gynephilic men and androphilic women. Men’s early and late processing of the stimuli was consistently gender-specific across both modalities. Women demonstrated gender-specificity only for the late processing (measured as dwell time) while watching static stimuli. Surprisingly, for the dynamic stimuli, they showed reversed patterns of gender specificity, gazing longer at nonpreferred targets than at preferred ones. For men of any orientation, cues regarding the gender and bodies of their preferred sexual partners are salient. No specific stimulus features appear uniquely salient for women—especially for androphilic women. However, no research has focused on testing the characteristics of sexual stimuli with regard to gender differences beyond the physical features of the depicted person's body (e.g. waist-to-hip ratio).

Visual attention studies show that some features of bottom-up stimulus processing are universally salient^25^. One of these features is movement, with fast movement attracting attention stronger than slow movement. However, there is a disparity in research regarding cognitive and genital responses to sexual stimuli. While most of the studies measuring genital reaction used film clips full of motion, the majority of studies using eye-movements and cognitive tasks employ motionless pictures. Still, pictures might just not be strong enough to elicit the same levels of affective reactions as videos (e.g.,^26^), possibly explaining gender-specific findings in female genital responses reported by Spape^8^ (although Sarlo and Buodo^27^ found gender-nonspecificity in female autonomic responses to erotic pictures). Additionally, film clips have naturally greater ecological validity and better reflect real-life erotic stimuli than still pictures^28^. Because of this disparity, we have no information about one of the major salient features in genital response-generating stimuli.

There is a specific motion connected with sexual intercourse in humans. This motion is easily recognizable because of a typical repetitive forward and backward hip movement, sometimes called copulatory movement (CM). Notably, it is present not only in humans but in all primates (and several other non-primate species), which might explain female genital responses to videos showing copulating primates^4,5^. CM is an inevitable result of regular penile thrusting during sexual intercourse, building up sexual arousal and eventually leading to ejaculation. It is virtually impossible for primates to copulate and avoid CM. As such, CM is an intuitive candidate for the universally recognizable feature of sexual stimuli and might be an important cue for the emergence of sexual response. Due to its gender-nonspecific nature—CM is present in all heterosexual, gay, and lesbian intercourse film clips—its role as a sexual cue might be more important in women—especially androphilic women—than in men.

The present study aimed to explore potentially different early attentional processing of CM in a sample of gynephilic men and androphilic women using eye-tracking.

## Study overview and hypothesis

Men's responses to sexually loaded stimuli are gender-specific, and women react in a non-specific way. Sexual response is triggered by recognizing specific sexual cues within the stimuli. CM is one of these cues present in nearly all video depictions of sexual intercourse, human and non-human. CM might represent an important cue for women resulting in genital responses to a wide variety of sexually loaded video stimuli. If this is the case, there should be clear preferential cognitive processing of CM in women but not in men, beginning with an attentional bias towards stimuli containing CM. We hypothesized that there would be an apparent bias in several indexes of early attentional processing (both for attentional bias and attentional capture), operationalized as latency to first fixation, duration of first fixation, position of first fixation, and dwell time towards stimuli containing CM, and that this bias will be larger in androphilic women than in gynephilic men.

## Methods and materials

### Participants

#### Power analysis

We conducted a priori power analysis to determine the required sample size. Using G*Power^29^ for repeated measures ANOVA with one within-subject and two between-subject factors, *1 − β* = 0.80, *α* = 0.05, a correlation between repeated measures of *r* = 0.50, and the effect size of *f* = 0.40, *n* = 56 will be needed for the purposes of the study. After adding 10% to account for possible data loss and rounding up, we aimed to collect data from at least 62 participants.

#### Sample description

Participants were recruited via social networks, the laboratory email list, and several university email lists. Initially, a sample of 74 participants was collected. Incomplete data (*n* = 9) and data from non-heterosexual participants (*n* = 5 participants scoring from “Equally heterosexual and homosexual” to “Exclusively homosexual” on the Kinsey scale) were excluded, leading to a total number of *n* = 60 participants.

All 29 men (*M*_*age*_ = 28.35 years, *SD* = 7.50) and 31 women (*M*_*age*_ = 27.55 years, *SD* = 7.49) had normal or corrected-to-normal vision and no diagnosed sexual or gynecological/urological issues. They were currently sexually active and not using any medications affecting their sexual functioning. Before the experiment, all participants signed an informed consent form approved by the Research Ethics Committee of the Faculty of Humanities, Charles University. The research was performed in accordance with the Declaration of Helsinki.

### Task development

Because of its dynamic nature, it is difficult to assess the attentional processing of CM. There are several ways of measuring the early attentional processes using cognitive tasks (i.e., dot-probe task^30^). These methods often suffer from poor validity and reliability estimates^31,32^. A great but technically challenging alternative is to use an eye-tracking device to measure eye movements as a proxy for measuring attention. Simultaneous presentation of two stimuli side by side and measuring behavioral indexes of attention works well for still stimuli^30^, and it might also work for dynamic stimuli. However, such a study has not yet been conducted. Moreover, commonly used video stimuli differ on many levels making the interpretation of behavioral data very difficult. For this purpose, we needed a new set of stimuli, homogeneous in multiple aspects but in the presence or absence of CM.

### Stimuli

The final film clip set consisted of four categories created by two factors: presence of CM (CM and non-CM) and content (heterosexual and lesbian). Based on the previous studies, we aimed to include the erotic film clips that were likely to elicit the strongest sexual response in both androphilic women and gynephilic men, that is, heterosexual and lesbian sex (e.g.,^4^). All film clips showed a sexual interaction between two partners. The films were further divided into penetrative and non-penetrative. The penetrative clips with CM showed penile-vaginal intercourse or penile-vaginal intercourse with the use of a strap-on dildo. The non-penetrative clips with CM included rubbing of genitals against each other with a typical hip thrusting but without penetration, or "scissoring". Penetrative film clips with no CM showed one partner fingering or giving a hand job to another partner. Lastly, the non-penetrative clips without CM depicted one partner rubbing and petting the genitals of another partner using hands in general. These categories assured a balanced spectrum of sexual intensity in both clips with and without CM. All categories were also balanced in terms of relative speed (Kruskal–Wallis one-way analysis of variance, *p* = 0.87). Each category consisted of four film clips, resulting in a final stimuli set of 16 unique short film clips.

### Stimuli development

Short film clips from freely available erotic films of heterosexual and lesbian pairs from the internet were acquired. Sexual content that corresponded with greater subjective sexual response in both androphilic women and gynephilic men was chosen, i.e., heterosexual and lesbian sex. The film clips depicted naked actors with no tattoos, jewelry, polish, or detailed objects in the background. Multiple alterations to films were made to reduce potential interfering factors like sound, face identification, or differences in color. All videos were muted, cropped, and centered on the genital area with parts of the thighs and torsos. Videos were transferred to a grayscale color range and balanced for its black and white levels.

Since there were no studies regarding early attention to movement, no clear stimulus duration could be clear from the available literature. However, based on a literature search in event-related potential (ERP) literature, attention bias literature, and the shortest time to first fixation (LFF) in eye-tracking studies, the shortest stimulus duration to capture the saliency effect was at least 500 ms. Given the slightly different movement speed of the stimuli, the final stimulus length was set to 2000 ms to capture two full iterations of CM at minimum.

Next, twenty independent raters (9 males, *M*_*age*_ = 26.10, *SD* = 4.64) were presented with all 47 film clips to evaluate two key aspects of the stimuli. First, since the conclusions of the study will be based on the difference in attention toward stimuli with and without copulatory movement, the presence/or absence of copulatory movement (operationalized as “pelvis thrusting typical for sexual intercourse”) in the film clips was evaluated. A simple single stimulus viewing paradigm was used with a question of whether the copulatory movement was present or absent. For most stimuli, the raters were in complete (100%) or partial (95–75%) concordance with the researchers’ initial assessment of copulatory movement presence/absence. However, for eight stimuli, a substantial number of raters (30–63%) did not agree with the initial assessment. These stimuli were removed from the stimulus set. Second, motion is perceptually salient^33^, and fast motion is more salient than slow motion^34^, so there was a need for homogenization of the stimuli in terms of movement speed. Subjective speed was evaluated by presenting all of our 47 stimuli (in the form of repeating gifs) in a randomized 7 × 7 grid (with no bottom corners). Raters were instructed to select the fastest stimuli. After they clicked on the gif, it disappeared. The task was repeated until no gifs remained on the screen. Each of the raters evaluated three randomized grids. Based on the data, six stimuli were removed because of a large standard deviation (*SD* > 6.5). From the remaining 33 film clips, 16 were selected based on their relative speed and content to match.

### Eye tracking

The participants were seated at a table with SR Research EyeLink 1000 Plus^35^ desktop-mounted monocular camera, approximately 70 cm away from a full high-definition monitor screen (1024 × 768 px resolution). Their head movements were constrained with a chin and forehead rest. At the beginning of the experimental procedure, a nine-point grid calibration was performed. Drift correction was used before each trial. The gaze samples were collected at 250 Hz. The data was collected from one eye only.

For the experiment, several indexes of early attention used in prior eye-tracking research dealing with sexually loaded stimuli^24,36,37^ were measured. Latency to first fixation (LFF) measures the time between the start of stimuli presentation and first fixation occurrence within the area of interest (AOI, defined as the entire area of either CM or non-CM stimulus, see Trial) and is used as an index of how quickly the stimulus captured interest of the participant. The position of first fixation (PFF) noted within which AOI landed the very first fixation of the trial was used as an index for preferential early attention and was presented as a percentage of first fixations for CM or non-CM stimuli. Both LFF and PFF should reflect a potential attentional bias towards CM, LFF by shorter mean latencies for first fixations in CM condition, and PFF by a larger percentage of all first fixations landing on CM film clip. Duration of first fixation (DFF) measures the duration of first fixation within the AOI and, together with the dwell time spent on AOI (DT, percentage of the 2000 ms), is used as an index of how captivating the stimulus was.

### Procedure

Participants were presented with a simple viewing task. Following calibration, participants were instructed to pay attention to the computer screen. The whole procedure took approximately 4 min to finish. After the assessment, participants were debriefed and gifted a small set of sex toys, condoms, and lubricants.

### Trial

**Figure 1:**
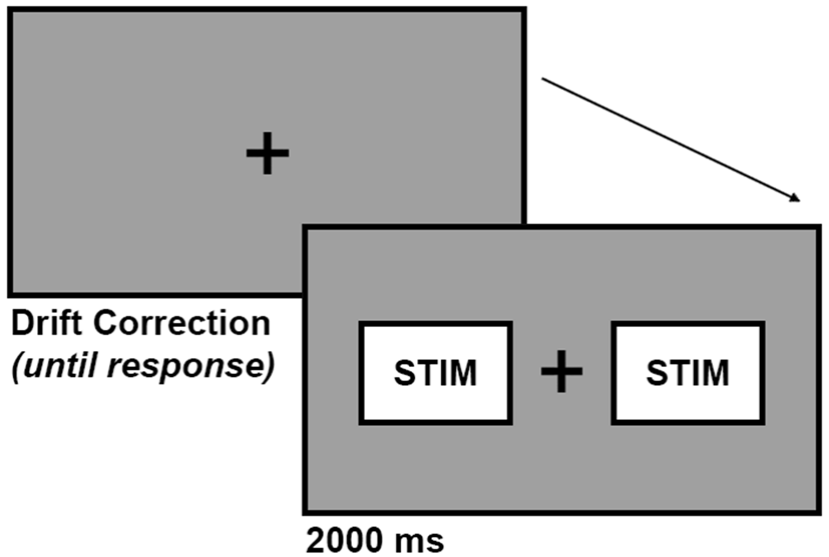
Trial structure.

Each trial consisted of a drift correction screen and a stimuli screen (see Fig. 1). The drift correction screen was presented as a small white target on a gray background (#999999). The participant would gaze directly at the target and confirm by pressing the space key. Next, two video stimuli appeared on the right and left sides of the screen (each video occupied 16.5% of the screen space with 480 × 270 px resolution). During the 2000 ms presentation of the stimuli, eye movements were recorded using EyeLink 1000 Plus (SR Research). After that, a new trial began with the appearance of a drift correction screen.

#### Counterbalancing

Each participant was presented with eight blocks of trials. Each block consisted of eight trials made from different combinations of CM and non-CM film clips (so that there was always one CM and one non-CM film clip in a single trial), resulting in 64 trials. Although thoroughly homogenized, the stimuli within a condition were not entirely similar, and presenting each CM stimulus with each non-CM stimulus would ensure that no single stimuli pairing would interfere with the overall results. It also helped to boost the number of trials significantly. There were two (A and B) block sequences of different trial combinations within blocks. Each participant was presented with one of these sequences. Randomly selected half of all the stimulus encounters were horizontally flipped in order to eliminate the effect of movement direction. Half of the CM stimuli appeared on the right side of the screen and half on the left. Block and trial order were completely randomized. The experimental procedure was created using Experiment Builder software (SR Research, version 2.2.245).

### Data analysis

Data were summarized using EyeLink Data Viewer 4.1.63^38^ and analyzed using R version 4.0.5^39^. CM and non-CM category means of LFF, DFF, DT, and PFF were calculated for each participant. No distinction was made for horizontally flipped stimuli or stimuli content in terms of heterosexual/lesbian behavior. Repeated measures ANOVA with one within-subject of movement (CM or non-CM) and two between-subject factors of sex (male or female) and order (A or B) was performed for all four dependent variables. Statistically significant results are reported as an estimation of effect size partial eta squared (*η*^*2*^_*p*_) for main effects and interactions and Cohen's *d* for post hoc comparisons. Strong and statistically significant (p < 0.001) lateral bias was revealed during the analysis of PFF, showing that most of the time, the participants looked at the left stimulus first (M = 85.60% for women and M = 79.40% for men). Moreover, eight participants first looked at the left side of the screen 100% of the time. However, since the results were not dramatically affected by their removal from the analysis, results with the full sample size are reported.

## Results

### Attentional bias

For the latency to first fixation (LFF), there were no main effects of movement, *F*(1, 56) = 1.18, *p* = 0.28, sex, *F*(1, 56) = 1.60, *p* = 0.21, or order, *F*(1, 56) = 0.04, *p* = 0.85. There was a statistically significant interaction effect of movement and sex, *F*(1, 56) = 4.06, *p* < 0.05, *η*^*2*^_*p*_ = 0.07. While there was a considerable gender difference in latency to first fixation to non-CM stimuli (see Table 1), post hoc tests with Bonferroni corrections did not reveal any statistically significant differences between the subgroups (all *p*s > 0.21). There was no interaction effect of movement and order, *F*(1, 56) = 1.11, *p* = 0.30.

**Table 1:** Mean (SD) values of all eye-tracking variables.

	Men (*n* = 29)		Women (*n* = 31)	
	CM	Non-CM	CM	Non-CM
LFF (ms)	650.74 (97.08)	620.61 (100.52)	661.67 (104.87)	671.16 (111.79)
PFF (%)	48.70 (4.80)	51.30 (4.80)	50.20 (3.90)	49.80 (2.90)
DFF (ms)	296.23 (52.50)	302.47 (77.33)	294.09 (59.48)	304.48 (59.32)
DT (%)	48.70 (8.70)	51.30 (8.70)	51.70 (9.10)	48.30 (9.10)

*LFF* latency to first fixation, *PFF* position of first fixation, *DFF* duration of first fixation, *DT* dwell time.

For the position of first fixation (PFF), there were no main effects of movement, *F*(1, 56) = 1.43, *p* = 0.24, sex, *F*(1, 56) = 0, *p* = 1, or order, *F*(1, 56) = 0, *p* = 1. There was no interaction effect of movement and sex, *F*(1, 56) = 3.82, *p* = 0.06. There was a statistically significant interaction effect of movement and order, *F*(1, 56) = 8.35, *p* < 0.01, *η*^*2*^_*p*_ = 0.13. Post hoc tests with Bonferroni corrections did show that in the second (B) ordering of trials, participants showed a larger preference for non-CM stimuli (*M* = 51.70%) than for CM stimuli (*M* = 48.30%; *d* = 0.93, *p* = 0.03). Generally, participants PFF were greater for CM stimuli in first (A) ordering (*M* = 50.80%) than in B ordering (*M* = 48.30%; *d* = 0.75, *p* = 0.03) and greater for non-CM stimuli in B ordering (*M* = 51.70%) than in A ordering (*M* = 49.20%; *d* = 0.75, *p* = 0.03). No other differences were found (*p* = 1).

### Attentional capture

For the duration of first fixation (DFF), there were no main effects of movement, *F*(1, 56) = 2.74, *p* = 0.10, sex, *F*(1, 56) = 0, *p* = 0.98, or order, *F*(1, 56) = 0.26, *p* = 0.61. There were no interaction effects of movement and sex, *F*(1, 56) = 0.14, *p* = 0.71, and movement and order, *F*(1, 56) = 0.06, *p* = 0.81.

For dwell time (DT) there were no main effects of movement, *F*(1, 56) = 0.03, *p* = 0.87, sex, *F*(1, 56) = 0, *p* = 1, or order, *F*(1, 56) = 0, *p* = 1. There were no interaction effects of movement and sex, *F*(1, 56) = 1.17, *p* = 0.29, and movement and order, *F*(1, 56) = 0.37, *p* = 0.55.

## Discussion

The present study aimed at assessing the potential sex differences in magnitude of attentional bias to erotic stimuli containing CM. Opposed to our hypothesis (i.e., the attentional bias of women towards stimuli containing CM), we found that both gynephilic men and androphilic women did not differ in attention bias to stimuli with CM. Also, we found a significant interaction of movement and sex in LFF, probably reflecting that men had considerably shorter latency to first fixations than women, but only in non-CM stimuli (see Table 1). Nevertheless, the difference was not statistically significant in the post hoc test after applying the Bonferroni correction. Another significant interaction occurred between movement and order in PFF. Namely, participants with the second ordering of trials were more likely to have their first fixation within the AOI of non-CM stimulus. The reversed pattern was observed for the first ordering of trials. No other main effect or interaction was found either in LFF or PFF. Given that the significance of the movement and sex interaction effect in LFF was already on the border of set significance testing, no statistically significant difference was found in pairwise comparisons, the relatively small effect sizes, and in the light of ordering having a larger effect on PFF than anything else, it can be safely assumed that found effects were most probably a result of measurement error.

Based on the IPM^19^ and studies by Chivers et al., we hypothesized that CM is an important sexual cue for women but not for men, and it should be reflected in cognitive processing. It appeared that the presence or absence of CM in erotic stimuli did not affect participants' early cognitive processing. We found no evidence to support the hypothesis that CM may be a critical feature of a sexually salient stimulus, given that early attentional processing was similar to sexual stimuli with or without such movement. Thus, the current study contributes to our understanding of what features may be fundamental to sexual stimuli.

However, another issue that was not accounted for in the planned statistical analysis arose during data processing. There was a strong lateral bias in participants' viewing strategy. In general, women looked first to the left film stimuli in 85.60% of trials and men in 79.40% of trials. Eight participants even used this viewing strategy exclusively, with no first fixations ever recorded on the right side of the screen. This is troubling because trials where the CM stimulus was on the left side made up exactly half of all trials, and consistent gazing upon one side of the screen will always result in a PFF of 50%. Although the results do not radically change after excluding these eight participants, considerable leftward bias in all participants probably skewed the data. Surprisingly, such bias is rarely reported in studies about the attentional processing of sexually loaded stimuli. In most cases, the researchers did not even test its presence in their analyses. It is thus possible that a leftward bias might have also been present in previous studies^24,36,37^. When reported, the bias is relatively small and does not interfere with the results^40^. Such large leftward bias, as found in our study, is exceptional and unprecedented in similar studies. Then again, this is the first time that a similar procedure has been applied to dynamic stimuli. The initial exploratory leftward bias might be triggered by the complexity of film clips^41^.

During the development of the stimuli, multiple methodological decisions were made to homogenize and balance the stimuli set in terms of color, laterality, content, and complexity. All these changes to the original source material in the name of methodological control certainly affected the ecological validity of the stimuli. Additionally, repeated exposures to the same (or very similar) stimuli probably impacted cognitive processing, quickly diminishing any novelty effect and potentially introducing an earlier onset of boredom and/or fatigue. The results of the present study should be interpreted strictly in the context of these methodological boundaries. Next, it would be beneficial to focus more on this potential hidden problem of eye-tracking studies and repeat the study in another cultural context to see if the bias persists or not. For example, a recent eye-tracking study on art and non-art imagery reported cultural differences in oculometric parameters, possibly related to different writing systems^42^.

While the occurrence of heavy leftward bias might erase any traces of attention bias, variables regarding attentional capture of the stimuli should not have been affected. Nevertheless, no main or interaction effects were found either in the duration of the first fixation (DFF) or the dwell time spent gazing at the stimulus (DT). Film clips with CM were in no way more captivating than those with no CM, and women did not differ from men in this indifference. Such results suggest that CM is not recognized differently in the erotic context.

## Conclusions

This study is the first to test directly the idea of copulatory movement as an important sexual cue responsible for gender-nonspecific sexual response patterns in women. We hypothesized that such cues would be cognitively salient and, therefore, favored in the early cognitive processing of the stimulus. While an unexpectedly large leftward bias prevented us from making definite conclusions regarding early attention bias to CM, the overall results show that CM (without other contextual cues) is not the most fundamental feature that captures attention and elicits a nonspecific response in androphilic women. Future research should include more diverse contextual cues to test the patterns of nonspecificity of visual attention in androphilic women.

Nonetheless, our sample was based on androphilic women and gynephilic men and did not test other important variables that could affect cognitive and visual processing. Due to these limitations, our findings are difficult to generalize. In future research, it would be advantageous to obtain more heterogeneous data encompassing variables such as participant age, cultural background, individual traits, or sexual preferences. It is possible that data across individuals of different social, cultural, and age groups could be divergent. Previous research already showed that individual variables such as sociosexuality, level of sexual desire, attitudes, sexual experiences, sexual disorders, or sexual excitation and inhibition propensity could also play an important role in visual processing (e.g.,^10,30,43–45^).

## Data Availability

The data has not been made available on a permanent third-party archive because of no consensus among the authors regarding the subject; requests for the data and materials can be sent to the corresponding author.

## References

[1] Chivers ML (2017). The specificity of women’s sexual response and its relationship with sexual orientations: A review and ten hypotheses. Arch. Sex. Behav..

[2] Chivers ML (2010). A brief update on the specificity of sexual arousal. Sex. Relatsh. Ther..

[3] Chivers ML, Rieger G, Latty E, Bailey JM (2004). A sex difference in the specificity of sexual arousal. Psychol. Sci..

[4] Chivers ML, Bailey JM (2005). A sex difference in features that elicit genital response. Biol. Psychol..

[5] Chivers ML, Seto MC, Blanchard R (2007). Gender and sexual orientation differences in sexual response to sexual activities versus gender of actors in sexual films. J. Pers. Soc. Psychol..

[6] Suschinsky KD, Lalumière ML, Chivers ML (2009). Sex differences in patterns of genital sexual arousal: Measurement artifacts or true phenomena?. Arch. Sex. Behav..

[7] Peterson ZD, Janssen E, Laan E (2010). Women’s sexual responses to heterosexual and lesbian erotica: The role of stimulus intensity, affective reaction, and sexual history. Arch. Sex. Behav..

[8] Spape J, Timmers AD, Yoon S, Ponseti J, Chivers ML (2014). Gender-specific genital and subjective sexual arousal to prepotent sexual features in heterosexual women and men. Biol. Psychol..

[9] Sawatsky ML, Dawson SJ, Lalumiere ML (2018). Genital lubrication: A cue-specific sexual response?. Biol. Psychol..

[10] Prause N, Janssen E, Hetrick WP (2008). Attention and emotional responses to sexual stimuli and their relationship to sexual desire. Arch. Sex. Behav..

[11] Shilhan J (2017). Attentional Processing of Visual Sexual Stimuli and The Concordia Sexual Image Dataset.

[12] Wierzba M (2015). Erotic subset for the Nencki Affective Picture System (NAPS ERO): Cross-sexual comparison study. Front. Psychol..

[13] Fromberger P, Jordan K, von Herder J, Steinkrauss H, Nemetschek R, Stolpmann G, Müller JL (2012). Initial orienting towards sexually relevant stimuli: Preliminary evidence from eye movement measures. Arch. Sex. Behav..

[14] Lykins AD, Meana M, Kambe G (2006). Detection of differential viewing patterns to erotic and non-erotic stimuli using eye-tracking methodology. Arch. Sex. Behav..

[15] Lykins AD, Meana M, Strauss GP (2008). Sex differences in visual attention to erotic and non-erotic stimuli. Arch. Sex. Behav..

[16] Baumeister RF (2000). Gender differences in erotic plasticity: The female sex drive as socially flexible and responsive. Psychol. Bull..

[17] Ganesan A, Morandini JS, Veldre A, Hsu KJ, Dar-Nimrod I (2020). Ethnic differences in visual attention to sexual stimuli among Asian and White heterosexual women and men. Pers. Individ. Differ..

[18] Suschinsky KD, Lalumière ML (2011). Prepared for anything? An investigation of female genital arousal in response to rape cues. Psychol. Sci..

[19] Janssen E, Everaerd W, Spiering M, Janssen J (2000). Automatic processes and the appraisal of sexual stimuli: Toward an information processing model of sexual arousal. J. Sex Res..

[20] Spiering M, Everaerd W, Janssen E (2003). Priming the sexual system: Implicit versus explicit activation. J. Sex Res..

[21] Spiering M, Everaerd W, Karsdorp P, Both S, Brauer M (2006). Nonconscious processing of sexual information: A generalization to women. J. Sex Res..

[22] de Jong DC (2009). The role of attention in sexual arousal: Implications for treatment of sexual dysfunction. J. Sex Res..

[23] Janssen E, Carpenter D, Graham CA (2003). Selecting films for sex research: Gender differences in erotic film preference. Arch. Sex. Behav..

[24] Dawson SJ, Chivers ML (2018). The effect of static versus dynamic stimuli on visual processing of sexual cues in androphilic women and gynephilic men. R. Soc. Open Sci..

[25] Connor CE, Egeth HE, Yantis S (2004). Visual attention: Bottom-up versus top-down. Curr. Biol..

[26] Boğa M, Koyuncu M, Kaça G, Bayazıt TO (2022). Comparison of emotion elicitation methods: 3 methods, 3 emotions, 3 measures. Curr. Psychol.

[27] Sarlo M, Buodo G (2017). To each its own? Gender differences in affective, autonomic, and behavioral responses to same-sex and opposite-sex visual sexual stimuli. Physiol. Behav..

[28] Gross JJ, Levenson RW (1995). Emotion elicitation using films. Cogn. Emot..

[29] Faul F, Erdfelder E (1992). GPOWER: A Priori-, Post Hoc-, and Compromise Power Analyses for MS-DOS [Computer Program].

[30] Novák O, Bártová K, Vagenknecht V, Klapilová K (2020). Attention bias and recognition of sexual images. Front. Psychol..

[31] Schmukle SC (2005). Unreliability of the dot probe task. Eur. J. Pers..

[32] Staugaard SR (2009). Reliability of two versions of the dot-probe task using photographic faces. Psychol. Sci. Q..

[33] Taylor SE, Fiske ST (1978). Salience, attention, and attribution: Top of the head phenomena. Adv. Exp. Soc. Psychol..

[34] Rosenholtz R (1999). A simple saliency model predicts a number of motion popout phenomena. Vision Res..

[35] SR Research Experiment Builder. (2019).

[36] Dawson SJ, Chivers ML (2016). Gender-specificity of initial and controlled visual attention to sexual stimuli in androphilic women and gynephilic men. PLoS One..

[37] Dawson SJ, Fretz KM, Chivers ML (2017). Visual attention patterns of women with androphilic and gynephilic sexual attractions. Arch. Sex. Behav..

[38] EyeLink Data Viewer. (2020).

[39] R Core Team R: A language and environment for statistical computing. *R Foundation for Statistical Computing, Vienna, Austria*. https://www.R-project.org/ (2021).

[40] Calvo MG, Lang PJ (2004). Gaze patterns when looking at emotional pictures: Motivationally biased attention. Motiv. Emot..

[41] Ossandón JP, Onat S, König P (2014). Spatial biases in viewing behavior. J. Vis..

[42] Brinkmann H, Mikuni J, Dare Z, Kawabata H, Leder H, Rosenberg R (2023). Cultural diversity in oculometric parameters when viewing art and non-art. Psychol. Aesthet. Creat. Arts..

[43] Brauer M, Van Leeuwen M, Janssen E, Newhouse SK, Heiman JR, Laan E (2012). Attentional and affective processing of sexual stimuli in women with hypoactive sexual desire disorder. Arch. Sex. Behav..

[44] Nolet K, Emond FC, Pfaus JG, Gagnon J, Rouleau JL (2021). Sexual attentional bias in young adult heterosexual men: Attention allocation following self-regulation. Arch. Sex. Behav..

[45] Aguiar S, Carvalho J, Carrito ML, Santos IM (2023). Automatic attention to sexual stimuli: Exploring the role of neuroticism and sexual excitation/inhibition through event-related potentials. J. Sex. Med..

